# Chapped Lips

**Published:** 1891-01

**Authors:** 


### CHAPPED LIPS.

A prominent authority highly praises this mixture for chapped lips:

℞ Tincture benzoini comp., f ℥ ij.

Glycerini, f ℥ vj. M.

Sig. Apply three or four times a day with a camel's hair pencil.

